# Improvements in Orthostatic Tolerance with Exercise Are Augmented by Heat Acclimation: A Randomized Controlled Trial

**DOI:** 10.1249/MSS.0000000000003355

**Published:** 2023-11-27

**Authors:** IAIN T. PARSONS, DANIEL SNAPE, MICHAEL J. STACEY, MATTHEW BARLOW, JOHN O’HARA, NICK GALL, PHIL CHOWIENCZYK, BARNEY WAINWRIGHT, DAVID R WOODS

**Affiliations:** 1 Research and Clinical Innovation, Royal Centre for Defence Medicine, Birmingham, UNITED KINGDOM; 2 School of Cardiovascular Medicine and Sciences, King’s College London, London, UNITED KINGDOM; 3 Carnegie School of Sport, Leeds Beckett University, Leeds, UNITED KINGDOM

**Keywords:** FAINTING, POTS, POSTURAL TACHYCARDIA, VASOVAGAL, PERFORMANCE ENHANCEMENT, SYNCOPE

## Abstract

**Introduction:**

Heat adaptation is protective against heat illness; however, its role in heat syncope, due to reflex mechanisms, has not been conclusively established. The aim of this study was to evaluate if heat acclimation (HA) was protective against heat syncope and to ascertain underlying physiological mechanisms.

**Methods:**

Twenty (15 males, 5 females) endurance-trained athletes were randomized to either 8 d of mixed active and passive HA (HEAT) or climatically temperate exercise (CONTROL). Before, and after, the interventions participants underwent a head up tilt (HUT) with graded lower body negative pressure (LBNP), in a thermal chamber (32.0 ± 0.3°C), continued until presyncope with measurement of cardiovascular parameters. Heat stress tests (HST) were performed to determine physiological and perceptual measures of HA.

**Results:**

There was a significant increase in orthostatic tolerance (OT), as measured by HUT/LBNP, in the HEAT group (preintervention; 28 ± 9 min, postintervention; 40 ± 7 min) compared with CONTROL (preintervention; 30 ± 8 mins, postintervention; 33 ± 5 min) (*P* = 0.01). Heat acclimation resulted in a significantly reduced peak and mean rectal and skin temperature (*P* < 0.01), peak heat rate (*P* < 0.003), thermal comfort (*P* < 0.04), and rating of perceived exertion (*P* < 0.02) during HST. There was a significantly increased plasma volume (PV) in the HEAT group in comparison to CONTROL (*P* = 0.03).

**Conclusions:**

Heat acclimation causes improvements in OT and is likely to be beneficial in patients with heat exacerbated reflex syncope. Heat acclimation–mediated PV expansion is a potential physiological mechanism underlying improved OT.

Heat stress, commonly in the form of elevated environmental temperatures, particularly when combined with orthostatic stress and/or exertion, can make considerable demands of the human cardiovascular system, which can manifest with collapse. In the absence of exercise, but in the presence of prolonged orthostasis, this is commonly known as heat syncope; a reflex syncope exacerbated by heat (1). Postexercise, a loss of the muscle pump, coupled with exertional hypohydration and increased venous pooling, leads to a reduction in central venous pressure and cardiac filling (2). Exercise mediated musculoskeletal vasodilation and a consequent reduction in systemic vascular resistance exacerbated by impairment of baroreflexes (3) coupled with altered postexertional thermoregulation (4) can lead to postexercise situational reflex syncope. Exercise-induced reflex syncope, as opposed to postexercise syncope, has been described (5) with the mechanism thought to be similar where there is blunted vasoconstriction of inactive skeletal muscle/viscera required to maintain blood pressure (BP) during pronounced vasodilation of exercising skeletal muscle (6). Endurance competitions are commonly undertaken in hot environmental conditions (7) and are associated with high rates of race withdrawal (8). Light headedness, faintness, dizziness, or collapse are common causes for presentation to medical facilities during, and after, endurance athletic events (9). The practical consequences of heat related incapacity encompasses the initial exclusion of life-threatening pathology such as heat illness or cardiac dysrhythmia. Further specialist investigation is often undertaken with consequential curtailment of recreational activities or occupational restriction.

Heat adaption through acclimatization or acclimation improves cardiovascular stability by maintaining cardiac output (CO), despite lowering heart rate (HR) (10), due to compensatory increases in stroke volume (SV) (11). The increase in SV is predominantly thought to be due to an increase in plasma volume (PV) (12). Acclimation to heat is associated with reduced incidence of postexertional syncope (13,14) during the first days of heat exposure (14) and may also reduce the incidence of reflex syncope generally, by improving orthostatic tolerance (OT). A series of studies addressing the effect of heat acclimatization on OT were performed in the 1970s by Shvartz et al. Initially, Shvartz demonstrated reduced fainting during a 20-min TT at 24°C and 40°C in 11 subjects during Summer compared with Winter (15). A further study was performed to determine the relationship of heat orthostatism and HA (16) in 18 Bantu males randomly split into three groups of six participants. This complex 10-d protocol involved groups working in the heat, working at room temperature as well as a resting group. Tilt-testing was performed, before and after work, at different temperatures. Those working in the heat developed improved OT to tilting in the heat. A further study was performed in 12 trained and 16 untrained men who underwent orthostatic stand tests before exercise, after exercise and after exercise in the heat. Tests were repeated in eight untrained participants after 8 d of HA. Trained individuals demonstrated better orthostatic responses than untrained (17). These studies demonstrate a consistent association of prior heat exposure with reduced syncope risk and improved OT. However, mechanistic insights were limited, as were clear aims and statistical analysis. The nonrandomized, often complex study designs leave the potential for confounders such as physical fitness (18) or hydration (19). Furthermore the majority of studies assessing the effect of heat adaptation on orthostatism have assessed OT in the immediate postexercise period (13,14,16,17) and while this appears to be protective with respect to postexercise collapse, the effect of HA on OT remote to the acute postexertional phase is less established. Further investigation of the effect of HA on OT is therefore merited.

The aim of this study was to further evaluate the potentially protective role of heat adaptation against heat syncope and to investigate underlying physiological mechanisms in athletic humans. We hypothesized that a heat acclimation (HA) regimen would improve OT in an athletic population. The primary endpoint was time to presyncope using an established model of syncope (20), performed in hot environmental conditions, before and after an intervention of HA or climatically temperate exercise.

## METHODS

### Overview

**FIGURE 1:**
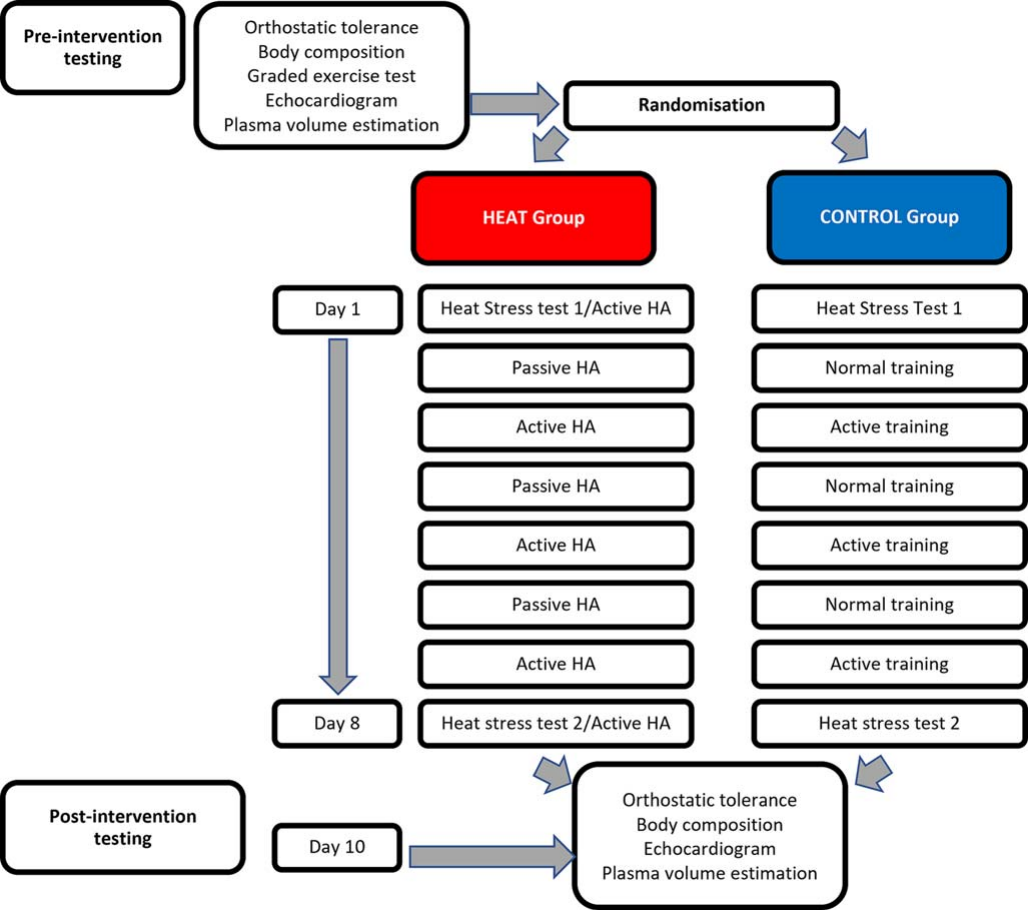
Schematic of the study protocol.

The study was registered with the ISRCTN registry (ISRCTN16534102). A schematic of the study protocol is provided at Figure 1. In brief, participants were randomized to either an active/passive combined HA group (HEAT) or control group (CONTROL). The latter performed equivalent work in thermoneutral conditions, compared with the active exercise-heat exposures of the HEAT group who also interspersed passive HA days in the place of the CONTROL groups’ usual exercise routines. A heat stress test (HST) was performed by both groups at baseline and then after the intervention, with OT assessed by head up tilt with lower body negative pressure (HUT/LBNP) and changes in PV and echocardiographic indices of cardiac function assessed concurrently.

### Participants

**FIGURE 2:**
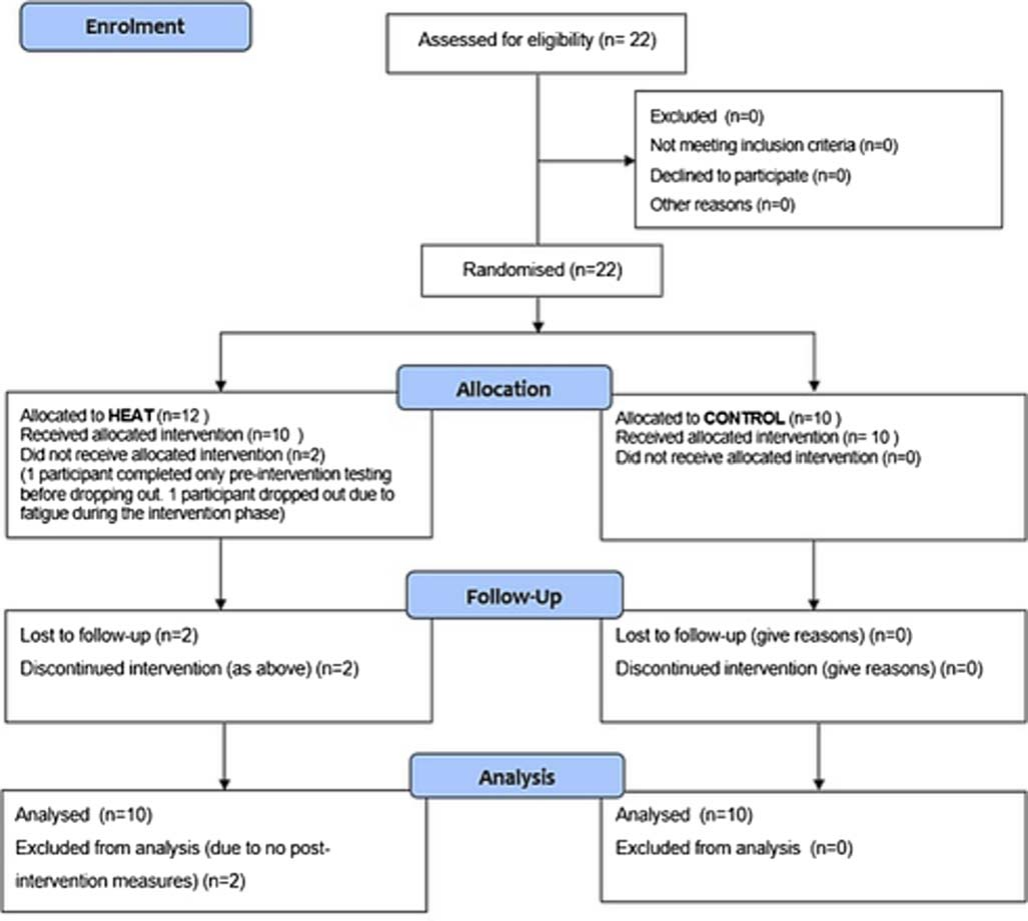
CONSORT Diagram describing the study enrolment, allocation, follow-up and analysis. Participants were randomized to one of two study interventions. We included data from 20 participants who completed the study protocol.

After approval by Leeds Beckett University Research Ethics Committee, 22 (17 males, five females) participants provided written informed consent with 20 participants completing the protocol (Fig. 1, Figure 2). Participants were fit and healthy, aged 18 to 44 yr. All participants were amateur endurance trained cyclists, or triathletes, classified as performance level 3 by training or V̇0_2peak_ (21). Each participant was unacclimated to the heat in that they had had no environmental heat exposure in the preceding 3 months. The trial was conducted over the course of one season (Spring 2021, Leeds, United Kingdom) to reduce seasonal acclimatization effects (22). Participants were instructed to avoid prolonged thermal exposures (saunas, steam rooms, hot baths, suntanning or exercising in hot environments) during the trial period. During the interval between initial measures and the intervention, participants were instructed to maintain their normal diet and training routines. All participants were asked to refrain from alcohol consumption at least 48 h before any testing and to avoid high intensity exercise in this period. Participants were asked to abstain from caffeine/stimulant containing drinks 6 h before testing.

### Preliminary Measurements

Measurements of stature (Seca, 220, Germany) were recorded. Body composition was determined by bioelectrical impedance (Tanita, Tokyo, Japan) preintervention and postintervention. A submaximal and maximal graded exercise test was performed preintervention to determine the participant's lactate threshold (LT), lactate turn point (LTP) (23), maximum HR (HR_max_), and maximal oxygen uptake consumption (V̇O_2max_). Respiratory gases were measured continuously using breath-by-breath on-line gas analysis (Cortex, Metalyser, 3B, Germany), calibrated following the manufacturer’s instructions.

### Heat Stress Testing and Heat Acclimation or Temperate Exercise Protocols

Participants were randomized using an online randomization tool (www.randomizer.org). All trials were scheduled at the same time of day to limit the confounding effect of circadian rhythm variation (24). Two hours before arrival at each experimental exposure, participants consumed 500 mL of water to ensure euhydration before each exposure. On arrival, participants provided a urine sample which was assessed for urine color, osmolality (Osmocheck, Vitech, Scientific Ltd, Japan) and specific gravity (Hand refractometer, Atago, Tokyo, Japan). Participants were deemed euhydrated when urine osmolality was <700 mOsmol·kg^−1^, specific gravity <1.020, and urine color <3 on an 8-point scale (25).

### Heat Stress Testing

To ascertain the physiological adaptations to HA, a HST was performed on day 1 and 8 in both the CONTOL and HEAT groups (Fig. 1). Female participants were scheduled to conduct the baseline HST and postintervention HST during the follicular phase of their menstrual cycle. The HST involved 45 min of submaximal fixed-intensity exercise on a cycle ergometer at 2.0 W·kg^−1^(Wattbike, Atom X, UK) in an environmental chamber (Sporting Edge (UK) Ltd, Basingstoke, UK), under hot, humid conditions (HST1: 32.0 ± 0.3°C, 72 ± 3% relative humidity [RH]; HST2: 32.1 ± 0.3°C, 71 ± 3% RH). After hydration assessment, rectal thermistor probe (4492, 400 series, Variohm, UK) connected to a data logger (SQ2020 2F8, Grant, UK) was self-inserted ~10 cm above the anal sphincter for the measurement of rectal temperature (T_RE_). Sweat patches (Tagaderm +Pad, 3 M, UK) were attached to the skin for whole body sweat sodium concentration estimation. Participants then entered the environmental chamber and rested in a seated position for 5 min before physiological (HR, T_RE_) and perceptual measures (perceived exertion [RPE] (26), thermal sensation (27) [TSS], and thermal comfort (28) [TC]) were recorded. Physiological strain index (PSI) was calculated during the HSTs from the equation as detailed by Moran et al. (29). During exercise HR was monitored continuously during all trials using telemetry (Polar, V800, Finland) and T_RE_ at 2.5-min intervals, while RPE, TSS, and TC were recorded at 5-min intervals. Fluid intake was restricted to room temperature water and consumed *ad libitum*. Towel-dried nude body mass (NBM) was measured before and after the trials and sweat rate (L·h^−1^) was estimated using changes in NBM from preexercise to postexercise periods, accounting for fluid intake and urine output but not insensible respiratory water and metabolic losses. Sweat patches were analyzed for Na^+^ concentration using a flame photometer (Jenway, PFP7 Flame Photometer, Essex, UK),

### HEAT Group

Those in the HEAT group completed an 8-d, combined active and passive HA protocol. This involved five isothermically clamped (30) (T_RE_ target 38.5–39.3°C) training sessions interspersed with three 30-minute treadmill runs (RPE = 13) in temperate conditions (20.9 ± 0.2°C, 58 ± 2% RH), followed by ≤40 min of hot-water immersion (HWI) (Fig. 1). For the HEAT group only, after completion of the HST, the exposure was extended to a total of 90 minutes to serve as an active HA day. During isothermic HA sessions participants trained using both intervals and steady-state cycling for 90 min·d^−1^ in the heat (32.2 ± 0.1°C, 71 ± 1% RH: 2.3 ± 0.1 W·kg^−1^). Each session involved a 10-min warm-up at the participants LT, followed by 10-minutes of high-intensity intervals over a 20-min period (1:1 work/rest) to get to target T_RE_ (intervals at participants LTP) followed by up to 60 min steady-state cycling to maintain an elevated T_RE_. Once the participant reached the target T_RE_, the power output (PO) was sustained, increased, or decreased and even rest periods allowed depending on the live T_RE_ response. HR and PO were recorded throughout, with perceptual measures at 10-minute intervals. Fluid intake was monitored during the trial period from pre-to-post bottle mass changes, measured to the nearest 0.1 g using weighing scales. For reasons of safety, trials in the heat were terminated if a core body temperature of 39.7°C was reached, ≥95% max HR or volitional exhaustion (31).

HWI sessions were performed on day 2, day 4, and day 7 of HA and were structured to align with athletes typical weekly running frequency when tapering for competition (32,33). Participants ingested a gastrointestinal pill (BodyCap, e-Celsius, France) ≥6 hours before the continuous measurement of gastrointestinal temperature (Tgi), synchronized to an external receiver (e-Viewer’ BodyCap, Caen, France) on arrival to the laboratory. Participants initially completed a 30-min treadmill run at a moderate-intensity (RPE = 13) under temperate laboratory conditions (20.9 ± 0.2°C, 58 ± 2% RH). This was immediately followed by up to 40-minutes HWI in a cryotherapy bath (CET CryoSpas, CryoSpa Sport, UK) submerged to the neck at a water temperature of (39.5 ± 0.1°C).

### CONTROL Group

The CONTROL group also completed a 10-min warm-up at the individuals LT, before completing high-intensity intervals over a 20-min period as described above, followed by up to 60-minutes steady state cycling. Thus, the CONTROL group (19.8 ± 0.3°C, 66 ± 6% RH: 2.3 ± 0.1 W·kg^−1^) completed training clamped to the same relative PO as the HEAT group. On days 2, 4, and 7 the CONTROL group performed their own normal unsupervised training.

### Preexposure and Postexposure Testing

#### Orthostatic tolerance

OT was measured preintervention and postintervention in a thermal chamber at 32.0 ± 0.3°C, 20 ± 3% RH. Orthostatic tolerance was measured using HUT/LBNP. Others have previously shown this technique to be reproducible and reliable for examining the effects of interventions aimed at improving OT (20,34,35). Participants rested in the supine position on the tilt table for 15 min to assess baseline cardiovascular parameters. After this, HUT was performed at time zero to an angle of 60° for 20 min. After this, while still tilted, LBNP was applied below the level of the iliac crest at −20 mm Hg for 10 minutes and was incrementally increased to −40 mm Hg and −60 mm Hg at 10-min intervals. The test was terminated by a blinded investigator at presyncope, defined as a systolic arterial pressure (SAP) below 80 mm Hg accompanied by symptoms of presyncope (e.g., light-headedness, blurred vision, dizziness). Orthostatic tolerance was defined as the time, in minutes, to presyncope.

#### Cardiovascular monitoring

Cardiovascular monitoring was performed using the volume-clamp method (Finapres Nova, Finapres Medical Systems, Amsterdam, The Netherlands). Continuous measures of SAP, diastolic arterial pressure (DAP) and mean arterial pressure (MAP) were obtained. Estimates of stroke volume index (SVI), cardiac index (CI) and total peripheral resistance index (TPRI) were obtained (Finapres Advanced Haemodynamics). HR and rhythm were monitored using a 3-lead electrocardiogram (Finapres ECG Module, Finapres Medical Systems, Amsterdam, The Netherlands).

#### Plasma volume estimation

Changes in PV (∆PV) were estimated following the equation described by Dill and Costill (36), from measured Hematocrit (Hct) and Hemoglobin (Hb) preintervention and postintervention. Whole blood collected into a plasma EDTA tube was inverted 8 to 10 times before immediately being analyzed for Hb and Hct using a hematology analyzer (Horiba, Pentra, ES 60, France), calibrated to the manufacturer’s instructions. Blood was collected with the participant lying at a 20° incline with a resting period of 30 minutes before collection. This was consistent preexposure and postexposure.

#### Echocardiography

Preintervention and postintervention participants underwent a resting transthoracic echocardiogram (TTE) (CX50, Phillips, Amsterdam, Netherlands) performed by a British Society of Echocardiography (BSE) accredited practitioner blinded to group. Echocardiography measures were performed in the resting partial left decubitus position with the couch head at 30° elevation (flat for subcostal views), in room temperature conditions, satisfying the requirements of the BSE minimum data set (37). Measures were averaged over Three heart beats. TTE was performed at a similar time of day to avoid potential diurnal variation. Left ventricular SV was calculated in two ways: 1) from the product of the velocity-time integral (cm) of the pulsed-wave Doppler in the left ventricular outflow tract (LVOT) and the LVOT cross sectional area (πr^2^; in cm^2^), determined by a TTE measurement of the LVOT in the parasternal long-axis view; 2) From the subtraction of the left ventricular end systolic volume (LVESV) from the left ventricular end diastolic volume (LVEDV) calculated using Simpson biplane method (37) in both two-chamber and four-chamber views, and then averaged. Atrial volumes were calculated using the area-length method (38). The septal and lateral mitral annular peak velocities were averaged.

### Data and Statistical Analyses

Measures were assessed for normality using the Shapiro–Wilk test before data analysis. For OT, echocardiographic values and physiological and perceptual measures of HA, preintervention and postintervention physiological measures were compared between the HEAT and CONTROL groups using a repeated measures two-way ANOVA. Volume-clamp derived cardiovascular measures at baseline were averaged and compared using a repeated measures two-way ANOVA. To ascertain if there were significant differences in cardiovascular values at presyncope a one-way repeated measures ANOVA was performed. Volume-clamp derived cardiovascular measures during HUT/LBNP were averaged to give a value for each 10-min phase of the HUT/LBNP protocol (HUT, 0–10 min; HUT, 10–20 min, and HUT+LBNP, 20–30 min) groups and were compared using a repeated measures 3-way ANOVA (mixed effects model to account for missing values where presyncope occurred earlier) with the dependent variable being the cardiovascular parameters and independent variables being test group (HEAT vs CONTROL), test intervention (preintervention or postintervention) and phase of TT. Linear regression was used to ascertain if resting OT correlated with improvements in OT after intervention in the HEAT and CONTROL groups. The repeatability of HUT/LBNP at 22–24°C is 1 ± 1 min (20). Accounting for a wider standard deviation with increased temperature, 10 participants per group would be able to detect a 4 ± 3 min difference between the two groups with a power of 0.8. A *P* value, adjusted for multiple comparisons where necessary, was significant at <0.05. All statistical analyses and graphs were performed on GraphPad Software, San Diego, CA.

## RESULTS

The physical characteristics of the two groups can be seen in Table 1. There was no significant difference in any Table 1 characteristic between the HEAT or CONTROL groups (*P* > 0.24). During HA and controlled exercise exposures there was no statistically significant difference in HR max (t-test) (HEAT: 80 ± 2%, CONTROL: 79 ± 4%, *P* = 0.49).

**TABLE 1:** Mean and standard deviation of baseline characteristics of study participants in the HEAT group; heat acclimation and CONTROL group; temperate exercise.

	CONTROL	HEAT
No. participants	10	10
Age (yr)	31 ± 9	28 ± 6
Sex (% female)	20%	30%
Height (cm)	179 ± 7	176 ± 9
Body surface area (m^2^)	1.94 ± 0.22	1.85 ± 0.16
V̇O_2max_ (mL·kg^−1^·min^−1^)	56.1 ± 11.3	55.9 ± 9.7
HR_max_ (/min)	183 ± 12	182 ± 12
Training Volume (h·wk^−1^)	12 ± 4	15 ± 7

The main effects of adaptions in HR, skin and core temperature, sweat rate and composition and perceptual measures of exertion and thermal stress during the HSTs preintervention and postintervention for the HEAT and CONTROL groups can be seen in Table 2. The hydration status before the HST and body composition changes preintervention and postintervention can also be seen in Table 2. In addition, the resting HR (adjusted *P* = 0.02), peak HR (adjusted *P* = 0.0007), resting T_RE_ (adjusted *P* = 0.0013), peak Tre (adjusted *P* = 0.01), mean T_RE_ (adjusted *P* = 0.0002), peak skin (adjusted *P* = 0.03), peak PSI (adjusted *P* = 0.02), mean PSI (adjusted *P* = 0.02), sweat loss (adjusted *P* = 0.02), total nonurinary fluid loss (adjusted *P* = 0.02), peak TC (adjusted *P* = 0.002), mean TC (adjusted *P* = 0.0002), peak RPE (adjusted *P* = 0.004) demonstrated significant differences in the HEAT group pre–post HA. The remaining HEAT measures in Table 2 were not significant (adjusted *P* > 0.08). In the CONTROL group pre–post intervention there was a significant increase in sweat sodium (adjusted *P* = 0.01) and Mean TC (adjusted *P* = 0.04). The remaining pre–post CONTROL measures in Table 2 were not significant (adjusted *P* > 0.08). On passive days (Fig. 1) participants in the HEAT group performed 35 ± 4, 36 ± 4, 39 ± 4 minutes of HWI respectively with a significant (*P* < 0.05) reduction in respective mean TC (3 ± 1, 3 ± 1, 2 ± 1) and mean HR (bpm) (107 ± 6, 104 ± 7, 100 ± 11). The mean core temperature during HWI was 38.6 ± 0.3°C.

**TABLE 2:** Physiological and perceptual measures during heat stress testing before, and after, HEAT acclimation (HEAT) or temperate exercise (CONTROL).

	CONTROL		HEAT		*P*
	Preintervention	Postintervention	Preintervention	Postintervention	
Heart Rate (/min)					
Resting heart rate	70 ± 11	70 ± 8	71 ± 9	62 ± 8	0.07
Peak heart rate	166 ± 16	168 ± 16	165 ± 18	152 ± 18	**0.003**
Temperature (°C)					
Resting temperature (RECTAL)	37.1 ± 0.2	37.1 ± 0.2	37.1 ± 0.2	36.8 ± 0.3	**0.006**
Peak temperature (rectal)	38.5 ± 0.3	38.6 ± 0.4	38.5 ± 0.2	38.2 ± 0.3	**0.01**
Mean temperature (rectal)	37.8 ± 0.2	37.9 ± 0.3	37.9 ± 0.2	37.6 ± 0.2	**0.0007**
Peak temperature (skin)	36.5 ± 0.5	36.6 ± 0.6	36.5 ± 0.4	36.1 ± 0.3	**0.03**
Mean temperature (Skin)	35.8 ± 0.6	36.1 ± 0.6	35.9 ± 0.4	35.6 ± 0.4	**0.006**
PSI					
Peak PSI	7.3 ± 0.7	7.5 ± 1.2	7.2 ± 1.2	6.3 ± 1.2	**0.02**
Mean PSI	5.3 ± 0.6	5.5 ± 0.8	5.4 ± 0.9	4.7 ± 0.9	**0.02**
Sweat					
Sweat loss (L)	1.82 ± 0.48	1.96 ± 0.65	1.67 ± 0.36	2.05 ± 0.54	0.24
Sweat sodium (mg·L^−1^)	35.3 ± 12.0	42.7 ± 10.8	44.1 ± 6.8	45.5 ± 5.8	0.10
Total nonurinary fluid loss (mL)	1363 ± 360	1476 ± 486	1249 ± 273	1535 ± 408	0.24
Perceptual markers					
Peak TSS	7.1 ± 0.8	6.8 ± 0.5	6.4 ± 1.2	6.1 ± 0.7	0.88
Mean TSS	6.4 ± 0.7	6.2 ± 0.6	6.0 ± 0.6	5.8 ± 0.7	0.67
Peak TC	4.2 ± 1.0	3.9 ± 0.9	3.6 ± 0.8	2.2 ± 1.0	**0.04**
Mean TC	3.3 ± 0.8	2.7 ± 0.8	2.9 ± 1.0	1.7 ± 1.0	0.09
Peak RPE	15.3 ± 3.0	15.2 ± 2.2	15.0 ± 2.9	12.7 ± 2.3	**0.03**
Mean RPE	13.2 ± 1.6	13.0 ± 1.5	12.4 ± 2.7	12.0 ± 1.8	0.81
Hydration status					
Urinary specific gravity	1.010 ± 0.008	1.011 ± 0.008	1.014 ± 0.008	1.012 ± 0.007	0.43
Urine Osmolarity (mOsmol·kg^−1^)	407 ± 284	443 ± 277	490 ± 307	478 ± 357	0.88
Change in body mass (%)	1.1 ± 0.5	1.2 ± 0.8	0.9 ± 0.5	1.5 ± 0.6	0.11
Fluid intake (mL)	523 ± 283	546 ± 318	589 ± 430	475 ± 209	0.31
Body composition					
Body mass (kg)	76.1 ± 15.0	75.4 ± 16.3	69.7 ± 10.3	71.8 ± 8.9	0.46
Body fat (%)	19.5 ± 4.7	18.4 ± 5.2	16.1 ± 4.5	14.7 ± 4.6	0.36
Total body water (%)	56.1 ± 2.8	56.3 ± 3.4	57.1 ± 3.4	58.6 ± 2.6	0.22

Bold denotes *P* value < 0.05.

Hydration measures and body composition values were measured before the heat stress test. *P* values relate to the main interaction, by two-way ANOVA, of HEAT and CONTROL groups, preintervention, and postintervention.

**FIGURE 3:**
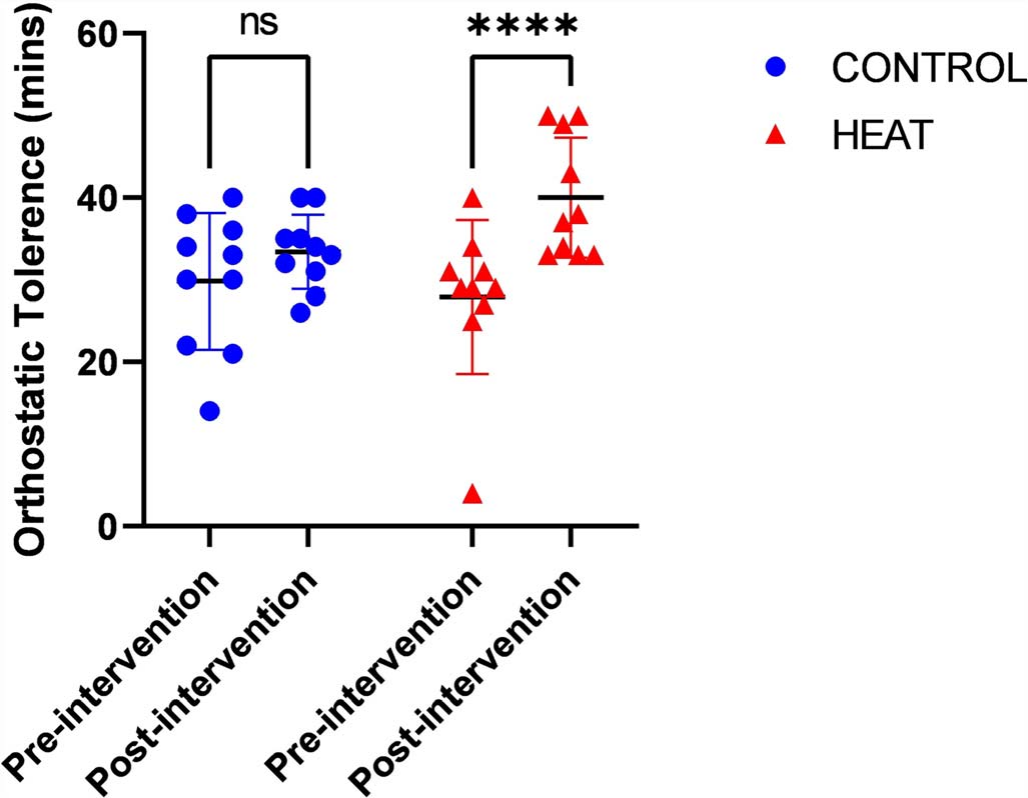
Individual participant OT measurements assessed by head up tilt with lower body negative pressure at baseline and after heat acclimation (HEAT) or temperate exercise (CONTROL).

One participant had a prior history of fainting who had the lowest OT (4 min) and was randomized to the HEAT group. In the HEAT group the mean time to presyncope at baseline was 28 ± 9 min and following HA was 40 ± 7 min. In the CONTROL group the mean time to presyncope at baseline was 30 ± 8 min and the mean time to presyncope after temperate exercise was 33 ± 5 min. There was a significant main effect in OT between HEAT and CONTROL preintervention and postintervention (F = 7.9, *P* = 0.01). *Post hoc* analysis within groups demonstrated a significant difference in the preintervention and postintervention in the HEAT group (adjusted *P* < 0.0001) but not CONTROL group (adjusted *P* = 0.21). The values of OT preintervention and postintervention can be seen in Figure 3. Simple linear regression demonstrated an inverse correlation between baseline OT and improvements in OT post intervention for both groups (R^2^ = 0.44, *P* = 0.001).

**FIGURE 4:**
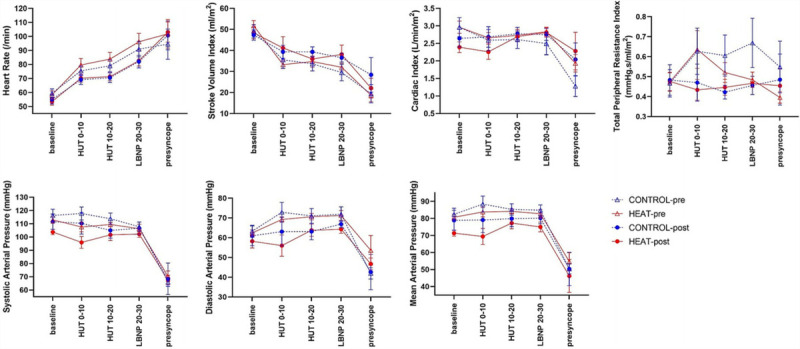
The mean and SD of cardiovascular parameters at baseline (before HUT), HUT 0-10mins, HUT 10-20mins, LBNP 20-30mins and presyncope for the CONTROL group before temperate exercise (CONTROL pre), posttemperate exercise (CONTROL-post), HEAT group before heat acclimation (HEAT-pre) and HEAT group post heat acclimation (HEAT-post).

For volume-clamp measures recorded during HUT/LBNP there was a main effect of time (preintervention to postintervention) for HR (CONTROL preintervention; 82 ± 15/min, HEAT preintervention; 86 ± 17/min, CONTROL postintervention; 74 ± 13/min, HEAT post intervention; 75 ± 11/min) (F = 20.0 *P* = 0.003), TPRI (CONTROL preintervention; 0.63 ± 0.30 mm Hg·s^−1^·mL^−1^·m^2^, HEAT preintervention; 0.55 ± 0.19 mm Hg·s^−1^·mL^−1^·m^2^, CONTROL postintervention; 0.45 ± 0.17 mm Hg·s^−1^·mL^−1^·m^2^, HEAT post intervention; 0.45 ± 0.13 mm Hg·s^−1^·mL^−1^·m^2^) (F = 4.80 *P* = 0.04) and SVI (CONTROL preintervention; 33 ± 11 mL·m^2^, HEAT preintervention; 33 ± 6 mL^−1^·m^2^, CONTROL postintervention; 38 ± 7 mL^−1^·m^2^, HEAT post intervention; 38 ± 11 mL^−1^·m^2^). (F = 0.4515 *P* < 0.05). There was no other main effect for any cardiovascular variable during LBNP, no main effect of group and no significant interaction between the HEAT and CONTROL groups preintervention and postintervention (*P* > 0.48). The mean and SD of volume-clamp derived cardiovascular parameters at baseline (before HUT), HUT 0 to 10 min, HUT 10 to 20 min, LBNP 20 to 30 min, and presyncope for the main cardiovascular parameters can be seen in Figure 4.

During supine rest in the heat chamber before head up tilt, there was a main effect of time (preintervention vs postintervention) (F = 6.30, *P* = 0.02) for HR (CONTROL preintervention; 59 ± 10/min, CONTROL post intervention; 55 ± 10/min, HEAT preintervention 58 ± 9/min, HEAT post intervention 53 ± 8/min and CI (CONTROL preintervention; 3.0 ± 0.9 L·min^−1^·m^2^, CONTROL post intervention; 2.7 ± 0.8 L·min^−1^·m^2^, HEAT preintervention; 3.0 ± 0.6 L·min^−1^·m^2^, HEAT post intervention; 2.4 ± 0.5 L·min^−1^·m^2^) (F = 9.15 *P* = 0.008) but there was no significant interaction between the HEAT and CONTROL when comparing interventions (*P* > 0.39). At presyncope, there was no significant difference (*P* > 0.32) between HEAT or CONTROL groups preintervention or postintervention, for any cardiovascular parameter.

We found a significant increase in PV in the HEAT group (9.6% ± 7.3) in comparison to CONTROL (−0.3% ± 11.1) (*P* = 0.03). Simple linear regression demonstrated a positive association between improvements in OT and % change in PV (R^2^ = 0.15) although this did not reach significance, *P* = 0.09). Resting echocardiographic data can be seen in Table 3.

**TABLE 3:** Mean and standard deviation of echocardiographic cardiovascular measures at rest before HUT/LBNP pre- and post-HEAT acclimation (HEAT) or pretemperate and posttemperate exercise (CONTROL).

		CONTROL		HEAT		*P*
		Mean (SD)		Mean (SD)		
		Preintervention	Postintervention	Preintervention	Postintervention	
LV volumes (indexed**)**						
End diastolic volume	(mL·m^2^)	82 ± 17	79 ± 15	86 ± 16	87 ± 13	0.11
End systolic volume	(mL·m^2^)	33 ± 7	32 ± 6	35 ± 8	33 ± 6	0.77
SVI (LVEDV-LVESV)	(mL·m^2^)	49 ± 11	47 ± 9	51 ± 9	54 ± 8	**0.01**
SVI (LVOT area x LVOT VTI)	(mL·m^2^)	49 ± 10	47 ± 8	49 ± 9	55 ± 9	**0.03**
**LV function**						
Ejection fraction	(%)	60 ± 3	60 ± 2	60 ± 3	62 ± 3	0.11
V_max_	cm·s^−1^	125 ± 28	115 ± 15	119 ± 17	127 ± 21	**0.03**
Cardiac Index	(L·min^−1^·m^2^)	2.6 ± 0.6	2.5 ± 0.6	2.8 ± 0.6	2.9 ± 0.6	0.35
**LV longitudinal function**						
MAPSE	(cm)	1.7 ± 0.3	1.7 ± 0.2	1.6 ± 0.2	1.6 ± 0.2	0.96
LV S′	(cm·s^−1^)	11.6 ± 1.9	10.9 ± 1.6	11.1 ± 1.7	11.5 ± 1.3	0.08
**RV longitudinal function**						
TAPSE	(cm)	2.7 ± 0.5	2.4 ± 0.3	2.6 ± 0.6	2.3 ± 0.6	0.96
RV S′	(cm)	13.3 ± 2.5	12.9 ± 2.9	12.8 ± 2.3	14.2 ± 1.9	0.18
**Diastolic function**						
E	(cm·s^−1^)	77 ± 17	73 ± 17	78 ± 11	83 ± 12	**0.02**
A	(cm·s^−1^)	43 ± 11	36 ± 10	46 ± 8	43 ± 12	0.62
E/A		1.86 ± 0.4	2.09 ± 0.6	1.72 ± 0.3	2.09 ± 0.7	0.62
Deceleration time	(ms)	220 ± 29	222 ± 47	240 ± 58	227 ± 44	0.50
e’	(cm·s^−1^)	15.7 ± 2.4	14.5 ± 2.2	15.6 ± 2.7	16.1 ± 2.2	**0.03**
a’	(cm·s^−1^)	10.2 ± 1.9	9.1 ± 2.0	9.5 ± 2.1	9.7 ± 2.1	0.11
E/e’		5.0 ± 1.3	5.1 ± 1.6	5.1 ± 0.9	5.3 ± 1.0	0.93
**Atrial volumes, cm**·**s**^−1^						
LA volume	(mL·m^2^)	33 ± 7	30 ± 10	27 ± 7	32 ± 7	**0.01**
RA Volume	(mL·m^2^)	32 ± 15	28 ± 9	26 ± 9	33 ± 11	**0.03**
**Inferior vena cava**						
IVC diameter expiration	(cm)	2.1 ± 0.3	2.3 ± 0.3	2.2 ± 0.7	2.2 ± 0.4	0.33
VC diameter inspiration	(cm)	1.3 ± 0.3	1.3 ± 0.6	1.3 ± 0.5	1.2 ± 0.4	0.92

Bold denotes *P* value < 0.05.

*P* values relate to the main interaction, by two-way ANOVA, of HEAT and CONTROL groups, preintervention, and postintervention.

MAPSE, mitral annular plane systolic excursion; LV, left ventricle; TAPSE, tricuspid annular plane systolic excursion; RV, right ventricle; E, early mitral inflow velocity; A, late mitral inflow velocity; e', mitral annular early diastolic velocity; a', annular late diastolic velocity; LA, left atrium; RA, right atrium; IVC, inferior vena cava.

## DISCUSSION

No prior study has, to our knowledge, attempted a randomized controlled trial to investigate mechanisms underlying improved orthostatism by HA, nor has a benefit to OT been quantified or mechanistically explored. This study confirms and builds on historical studies performed in the 1970s by Shvartz et al. The initial study (15) demonstrated reduced fainting during a 20-min TT at 24°C and 40°C in 11 subjects during summer compared with winter. This nonrandomized, nonblinded protocol is susceptible to confounding factors that could explain decreased fainting in summer than winter e.g., performing more exercise (39) in summer months or increasing salt intake (40). Relative humidity was not controlled for during TT and varied seasonally. Mechanistic interpretations were limited and while a 10-min bicycle ergometer was performed after each test this did not reveal significant differences between seasons in HR measured in only in the final minute of the test. A further study was performed by Shvartz et al. to determine the relationship of heat orthostatism and HA (16) in 18 Bantu males randomly split into three groups of six participants. The complex 10-d experimental protocol involved two TT performed either side of an exposure. Group A worked in the heat with the first TT in 21°C and second TT performed “in heat.” Group B worked at room temperature and were tilted at room temperature. Group C rested in the heat but were tilted as per group A. Only one fainting episode occurred in group B with no fainting episodes in group C. Group A showed a reduction (without significance testing) in fainting over the 9 d. On day 10, groups B and C underwent the same protocol as group A. Here there were five fainting episodes in group C and one fainting episode in group B. This was despite group C demonstrating decreases in rectal temperature. This would somewhat suggest that it was exercise rather than heat acclimatization that was the most causative factor in improving OT. Group A may have seen a reduction in TT-induced syncope by tilt-training (41). A further study by Shvartz et al. assessed OT, this time using an active stand test, 20 min before exercise, after 60 min of exercise with a 40-W load, after 15 min of exercise at an 80 W load, and after 3 h of exercise with a 40 W load in 39.4°C, in 12 trained and 16 untrained young males. Eight of the untrained subjects were retested in the five orthostatic tests after 8 d of HA. The number of fainting participants in the orthostatic tests administered before exercise, after exercise at 40 W and 80 W, and after exercise in heat, were 3, 4, 6, and 13, respectively. The trained subjects were less likely to faint than untrained (10% vs 25% respectively) in pooled analysis. HA improved tolerance to fainting in the untrained but it is again difficult to ascertain whether there was a training effect. Furthermore, in many of these studies the orthostatic testing was performed post exercise (13,14,16,17). In the postexercise phase, the widespread vasodilation and consequent reduced TPR in conjunction with loss of the muscle pump can result in postexercise hypotension and collapse. However, numerous other factors such as heat, dehydration and potentially participant muscle mass also play a role (9). Indeed, there is data to indicate that exercise in the heat, regardless of acclimation status, causes profound postexercise hypotensive responses (42) despite orthostatic responses improving with HA (13,14,16,17).

Our study was unique in that the measure of OT was performed remote to exercise exposure. We were able to conclusively demonstrate using a randomized, controlled, single blinded design, that HA significantly improves OT, as assessed by HUT/LBNP, to a substantial extent (~43% absolute gain, 33% relative gain versus temperate exposures). This investigation also offers the fresh insight that HA-associated echocardiographic changes—namely significantly increased atrial volumes, SVI, V_max_, E and e’, plus a tendency to increased LVEDV—arising with medium term HA (43) remain consistent with those observed across a 5-d HA protocol and do indeed associate with enhanced PV status, as previously hypothesized (44). The increase in SV and atrial volumes with HA would support an increase in preload occurring secondary to increased PV. The significant increase in V_max_ was likely mediated by increased preload, resulting in increased left ventricle (LV) contractility as a function of the Frank-Starling length-tension relationship. The relatively elevated E (rapid early diastolic filling) in HEAT was consistent with the LV filling effects of increased PV. With a concomitant rise in the speed of early LV relaxation (e’) during diastole, reflecting increased ventricular compliance or reduced LV stiffness, the heart would be potentiated to convert an elevated PV, and consequently increased LV preload, without elevation of LV end-diastolic pressure. These parallel changes in E and e’ explain the minimal change observed in E/e’, a surrogate of LV filling pressure and a predictor of LA pressure (45). However, while we found a significant increase in PV in the HEAT group compared to CONTROL, on regression analysis we were unable to find a significant (*P* = 0.09) correlation between changes in PV and changes in OT following intervention. A more accurate method of measuring PV, e.g., using a dye dilution method or radioactive tracer (46) may have demonstrated this and indeed PV has been shown to be positively correlated to OT in previous studies (47). These data point to the ways in which HA may protect CO when baroreceptor regulated responses would otherwise be overwhelmed by heat stress (48) and therefore reduce the risks of syncope, co-incident with improving performance capacity relative to initial heat exposure (32) (33) (34–36).

The HA regimen selected for this study represents those likely to be selected for athletes preparing for competition (43,49). Here the benefits of deescalated training volumes must be balanced against any gains associated with heat adaptation to environmental conditions. While our data derive from sub-elite athletes, these individuals were nevertheless well-trained and accord with other groups of similar aerobic training status who face similar challenges, for example military personnel. Using physically trained participants and utilizing a control group was important as exercise, albeit more slowly (50), induces many of the cardiovascular changes seen in HA (51) including lower HR, increased PV and increased OT (39). Extending our findings beyond the study population, we found that the lower the baseline OT, the greater the improvements in OT after HA or controlled temperate exercise, suggesting that heat adaptation would be of value to individuals prone to heat-related syncope, over and above that of exercise, to rapidly improve OT. While we used a relatively robust medium-term HA protocol this was to demonstrate a difference of HA above that of exercise in a physically fit group and we would anticipate that a less robust heat acclimatization regimen would result in beneficial results in a less physically fit patient population. A mixed-heat acclimatization regimen is however less fatiguing for nonathletic patients attempting to prevent reflex syncope, without the use of medications, with HWI easily performed using jacuzzi baths or even substituted for steam rooms or saunas (52,53) if an adequate elevation in skin temperature is achieved (54). Preliminary echocardiographic data (44) coupled with these data suggests improvements in diastolic function with HA above that of exercise alone. Exercise, regardless of weight loss, reduces occurrence and symptoms of atrial fibrillation and this is, in part, thought to mediated by improvements in diastolic dysfunction (55). Exercise is known to improve diastolic function even in the setting of heart failure (56). Heat acclimation, in addition to exercise, may serve as a useful additional means to prevent or reduce atrial fibrillation (where the cause is not due to endurance exercise) particularly in patients with diastolic dysfunction. This requires further study in a suitable patient population. While we did not measure OT in the immediate phase postexertion, it would appear from previous work that HA would be protective against postexercise collapse (13,14,16,17).

There are several potential limitations to this study. We had unequal numbers of males and females in the study which may affect generalizability of the study. While we have presented data suggesting the mechanism of increased OT after HA, compared to temperate exercise, is due to PV expansion we acknowledge that this is associative but may not be causative. Other mechanisms which could potentially contribute to the relative improvements in OT seen in this study include a reduction in sympathetic activity. The acquisition of the HA phenotype has been shown to associate with relative sympathetic withdrawal in response to heat stress (57). A further study using microneurography measures, catecholamine levels along with noninvasive measures of sympathetic activity during tilt, pre- and post-HA, would be valuable. To limit participant visits we opted to incorporate heat stress testing into the first and final acclimation days, which may have reduced the magnitude of results obtained during heat stress testing. However, significant responses were still demonstrated indicating HA had occurred. Hypohydration is a possible confounder in the measurement of OT however we recognized no difference in hydration markers during the HST. The avoidance of dehydration also does not fully attenuate the effect of the heat (58). We also did not measure the CONTROL groups training burden on the passive days in the HEAT group however cardiovascular strain was similar between groups. Echocardiography has significant inter-observer and intra-observer variability particularly in the measurement of LV volumes (59) which may explain why we were unable to demonstrate a significant increase in LVEDV in the HEAT group, in comparison to CONTROL, during this study in comparison to previous (44). However, we used the same echocardiographer to limit interobserver variability, who was blinded to the participants intervention. We were unable to demonstrate any significant differences in the volume-clamp data at rest nor during HUT/LBNP between the HEAT and CONTROL groups. This may be partly due to the suboptimal performance of the volume-clamp in the heat chamber while all echocardiographic measures were taken in a temperate environment at rest. We are confident however that the increased OT in the HEAT group is not a function of this group having a lower baseline OT as there was no significant difference in baseline OT between the HEAT and CONTROL groups.

## CONCLUSIONS

In summary HA causes improvements in OT, in comparison to exercise alone, and is likely to be beneficial in patients with orthostatic intolerance particularly when exacerbated by heat stress. PV expansion may be a contributory mechanism underlying improvements in OT after HA.

## Declarations

Funding: This study was supported by the Ministry of Defense

Conflicts of interest/Competing interests: Not applicable. The results of the study are presented clearly, honestly, and without fabrication, falsification, or inappropriate data manipulation. The results of the present study do not constitute endorsement by the American College of Sports Medicine.

Data Availability Statement: Data available on request

Ethics approval: Ethical approval was obtained from the Leeds Beckett University

Consent for publication: obtained

Authors' contributions: I.T.P. conceived the study, collected results, performed data analysis, and drafted the manuscript. D.S. collected results and reviewed the manuscript. M.S., D.W., J.O.H., B.W. critically edited the manuscript and contributed to the content. P.C. and N.G. critically edited the manuscript and reviewed the content. D.W. was responsible for the overarching review of the content.

Registration: ISRCTN16534102

Conflict of Interest and Funding Source: This study was supported by the Ministry of Defense. The authors have to conflicts of interest to declare.
